# P043 Hiding in plain sight - immunotherapy’s “giant” complication

**DOI:** 10.1093/rap/rkag113.073

**Published:** 2026-11-03

**Authors:** Samuel McCormick, Ryan Hackett

**Affiliations:** Belfast Health and Social Care Trust, Belfast, United Kingdom; Belfast Health and Social Care Trust, Belfast, United Kingdom

## Abstract

**Introduction:**

Immune checkpoint inhibitors have emerged as a major advancement in cancer treatment, harnessing the body’s immune system to identify and eliminate malignant cells. They are used in the treatment of a wide range of cancers with remarkable results. Despite their clinical benefits, they can induce immune-related adverse events resulting from nonspecific activation of the immune system, affecting a wide range of organ systems. This case is an example of this with rheumatological consequences.

**Case description:**

A 75-year-old male was diagnosed with epithelioid mesothelioma in August 2025. He started palliative immunotherapy in October 2025 with a combination of ipilimumab and nivolumab.

The patient completed 3 cycles without issue before presenting with complications during his 4th cycle in March 2026. He initially presented to the oncology assessment unit complaining of jaw claudication, scalp sensitivity and a throbbing temporal headache. Blood tests showed an elevated ESR of 94 and CRP of 83. The patient was discharged on prednisolone 40mg OD with follow-up at the immuno-oncology steroid clinic. Immunotherapy was placed on hold.

The patient attended for review one week later, complaining of temporal headache and new blurred vision with floaters. Temporal pulses were weak bilaterally without tenderness. Ophthalmology review showed bilateral visual field loss and optic disc swelling consistent with bilateral AION. The patient was initially treated with IV methylprednisolone. Rheumatology review advised tapering the prednisolone regime in combination with methotrexate.

Temporal artery ultrasound halo and compression signs in bilateral common superficial temporal arteries were present. Frontal and parietal branches on both sides also showed evidence of GCA. Temporal artery biopsy showed inflammation in the Tunica media and adventitia. Giant cells were present. This is consistent with temporal arteritis.

Interval CT scanning of his mesothelioma has shown complete response to treatment with no visible disease remaining. Therefore, oncology has agreed a watch and wait strategy. The patient was reviewed by rheumatology in May 2026 and all symptoms have resolved completely. He remains on methotrexate and a slow weaning course of prednisolone in conjunction with ophthalmology.

**Discussion:**

This case highlights a rare but clinically significant immune-related adverse event associated with immune checkpoint inhibitor (ICI) therapy. The diagnosis of GCA was considered due to the patient’s clinical presentation and inflammatory markers in the context of recent ICI exposure. Prompt recognition was essential given the risk of irreversible visual loss. High-dose corticosteroid therapy was initiated in accordance with established GCA management principles, balancing the need for rapid immunosuppression against the potential impact on antitumour effect.

This case emphasises the importance of maintaining a broad differential diagnosis when assessing patients receiving ICIs. Symptoms that may initially appear nonspecific can represent immune-mediated toxicity rather than disease progression, infection, or treatment-related side effects. Early multidisciplinary involvement between oncology, rheumatology, and ophthalmology was vital in establishing the diagnosis and guiding management decisions.

The case is particularly interesting because ICI-induced GCA remains uncommon and the underlying mechanisms are not fully understood. As the use of ICIs continues to expand across multiple malignancies, clinicians are likely to encounter an increasing number of rare autoimmune complications. Reporting such cases contributes to the growing body of evidence regarding their presentation, diagnosis, and optimal management.

Several questions remain unanswered. It is unclear whether patients who develop ICI-induced vasculitis have identifiable risk factors or whether certain agents confer a greater risk than others. The optimal duration of immunosuppressive therapy and the safety of re-challenging patients with ICIs after resolution of GCA also remain areas of ongoing debate. Further research is required to better define management strategies while preserving oncological outcomes.

**Key learning points:**

• Immune checkpoint inhibitors can cause wide-ranging immune-related adverse events, including rheumatological complications like GCA.

• New-onset headache, scalp tenderness, jaw claudication or visual symptoms in patients receiving ICIs should prompt urgent evaluation for GCA given the risk of irreversible visual loss.

• Diagnosis should follow established GCA pathways such as vascular imaging and temporal artery biopsy.

• Management of ICI-induced GCA requires multi-disciplinary collaboration to balance the need for immunosuppression with maintenance of antitumour efficacy.

Learning objectives:

• To enhance my understanding of the diagnosis and management of rheumatic immune-related adverse events, particularly ICI-induced GCA.

• To keep up to date with recent research and evolving guidelines on immune checkpoint toxicity and vasculitis.

• To gain insight into current evidence for balancing immunosuppressive therapy with oncological treatment in patients who develop immune-related adverse events.

• To strengthen multidisciplinary approaches between rheumatology, oncology and ophthalmology to optimise outcomes in patients with immune-related adverse events from ICIs.

